# Death circumstances of older adults with cannabinoid exposures

**DOI:** 10.1093/geroni/igaf122.2261

**Published:** 2025-12-31

**Authors:** Armiel Suriaga

**Affiliations:** Florida Atlantic University, Boca Raton, Florida, United States

## Abstract

Results from the 2021 and 2022 National Drug Use and Health Survey reported that 7.7 million older adults (60+) used cannabis. Reasons for cannabis use were to feel good, relax, improve sleep, and pain relief. However, studies on the long-term effects of cannabis in the older population remain limited. Our cross-sectional study aimed to report the characteristics and the death circumstances of people (65+) with cannabinoid exposures as determined by the medical examiners through autopsy and toxicology results. We used descriptive statistics to examine the de-identified data from the Florida Department of Law Enforcement (2020-2022). There were 10,851 (23.37%) out of 46,437 cases with cannabinoid exposures; 576 (5.31%) were older adults. 482 (83.68%) were males, whites (496 or 86.11%), and 576 (94%) happened in urban counties. 213 used a single drug, while 363 (63.02%) had polydrug use. 180 older decedents had cardiac-related conditions, 57 with respiratory-related conditions, and 14 with diabetes. The manner of death were accidents (n = 247 or 42.88%), homicide (n = 12 or 2.08%), natural cause (n = 177 or 30.73%), suicide (n = 133 or 23.09%), and undetermined cause (n = 7 or 1.22%). 115 older adults with cannabinoid exposures died from intoxication, while 28 (4.64%) from motor vehicular accidents. Using univariate logistic regression, cannabis users had 60% odds of dying from intoxication compared to non-users, OR=.604, 95% CI, .57-.63, p < 0.001. After adjusting for confounders, the odds of experiencing toxicity decreased, OR=.35, 95% CI, .33-.37, p < 0.001. Our study provided evidence of the harm from cannabinoid exposures, underscoring the need for tailored interventions for public safety.

